# Analysis of Myelodysplastic Neoplasia: Epidemiological and Biological Characteristics Based on 20 Clinical Cases and a Literature Review

**DOI:** 10.7759/cureus.111501

**Published:** 2026-06-25

**Authors:** Fatiha Bousnina, Samira Laghzaoui, Mohammed Bensalah, Assya Khermach, Rachid Seddik

**Affiliations:** 1 Department of Hematology, Mohammed VI University Hospital, Faculty of Medicine and Pharmacy, Mohammed First University, Oujda, MAR

**Keywords:** bone marrow aspirate, classification, complete blood count, malignant hematopathy, myelodysplastic neoplasia

## Abstract

Myelodysplastic neoplasia (MDS) is a hematological malignancy characterized by inefficient hematopoiesis, responsible for quantitative and qualitative abnormalities of the three myeloid cell lineages, classically revealed by peripheral cytopenias and an increased risk of transformation into acute myeloid leukemia (AML). The diagnosis of these conditions is sometimes difficult, complex, and time-consuming, potentially requiring multiple examinations, relying primarily on complete blood count (CBC) data and the results of semi-quantitative and qualitative analysis of the bone marrow aspirate. Our objective through this study is to determine the epidemiological and biological profile of MDS. We included 20 patients presenting with MDS diagnosed on CBC and bone marrow aspirate analysis. Within our population, male predominance was observed, with a mean age of 61 years. Biologically, the CBC demonstrated the presence of anemia in 83.33% of cases with a mean hemoglobin concentration of 7.92 g/dl. Furthermore, thrombocytopenia was detected in 55.55%, while neutropenia was observed in only 44.44% of patients. Using the World Health Organization (WHO) 2022 classification, the entity of MDS with few blasts and multilineage dysplasia (MDS-LB-MLD) was the most frequently detected in our study (35%). According to cytogenetic analysis results, a single case of 5q- syndrome and one case of chromosome 7q deletion were identified among the 13 studies performed on all patients. Recently, MDS has attracted increased attention, particularly regarding diagnostic, prognostic, and therapeutic criteria.

## Introduction

The term myelodysplastic neoplasia (MDS) designates a group of clonal disorders affecting hematopoietic stem cells. Typically acquired and rarely congenital, these hematological malignancies are characterized by ineffective hematopoiesis, leading to peripheral cytopenias and qualitative abnormalities across the three myeloid cell lineages, with an increased risk of transformation into acute myeloid leukemia (AML) [1,2]. The diagnosis of these conditions is complex and often requires a multi-step approach, relying primarily on complete blood count (CBC) data and comprehensive bone marrow aspirate analysis [3]. While the prognosis of these conditions was historically unfavorable, recent diagnostic advancements have significantly improved patient stratification [1].

The primary objective of this study is to characterize the clinical, hematological, and cytomorphological profile of patients diagnosed with MDS at our center. Our secondary objectives are twofold: first, to classify our patient cohort according to the 2022 World Health Organization (WHO) diagnostic criteria, and second, to assess the prognostic risk of these patients using the Revised International Prognostic Scoring System (IPSS-R), incorporating available cytogenetic data [4,5].

## Materials and methods

We present a retrospective, observational, and descriptive study conducted over three years, from October 2021 to October 2024, within the Hematology Department of the Mohammed VI University Hospital in Oujda. During this period, 29 bone marrow aspirate examinations were performed in our laboratory for 20 unique patients diagnosed with MDS. The difference between the number of samples (n=29) and the number of patients (n=20) is due to follow-up bone marrow aspirates performed in some patients to monitor disease progression. For the clinical and biological baseline analysis, only the initial diagnostic bone marrow sample was utilized for each patient, ensuring the independence of our data and avoiding duplication bias. Patients were identified based on MDS documentation through CBC and bone marrow analysis, with diagnoses established in strict accordance with the 2022 WHO classification criteria.

Sociodemographic, clinical, and laboratory data for each patient were collected, including blood count results obtained via the hospital information system (Hosix). Compliance with the pre-analytical protocol was systematically verified upon receipt of the bone marrow aspirate. Each sample underwent an initial inspection, and all relevant information, including patient identity, puncture site, and clinical data, was recorded. The bone marrow aspirate slides were stained using the May-Grünwald-Giemsa (MGG) technique. This is a standard hematological staining method used to visualize cellular morphology, allowing for the precise identification of dysplastic changes and quantitative assessment of myeloid precursors in bone marrow specimens. The staining procedure was as follows: slides were immersed in undiluted May-Grünwald solution for five minutes, followed by a 25-minute incubation in a 1/10 diluted Giemsa solution. This optimized protocol ensures the high-quality visualization of cellular morphology required for the accurate identification of dysplastic features across the three myeloid lineages, in accordance with the 2022 WHO classification criteria.

All these data, particularly those from the CBC and bone marrow aspiration, were used to establish the diagnosis and classification of MDS according to the 2022 WHO diagnostic criteria. For all patients, the diagnosis was established or re-evaluated to ensure strict adherence to these criteria. This re-evaluation was carried out by two independent hematologists, thereby ensuring the consistency and accuracy of the diagnosis. For cases diagnosed prior to 2022, this systematic re-evaluation process was particularly crucial. Significant dysplasia was defined as the presence of morphological abnormalities in at least 10% of cells, assessed on a count of at least 200 cells for the erythroid and granulocytic lineages and 30 cells for the megakaryocytic lineage. Patients with vitamin B9, vitamin B12, and/or iron deficiency, as well as those with secondary MDS, were excluded from the study.

Regarding laboratory reproducibility, all analyses were performed in a clinical setting following standard internal quality control protocols. However, we acknowledge that the study's reproducibility is partially limited by the retrospective nature of data collection, the lack of systematic independent inter-observer assessment for morphological analysis, and the non-availability of comprehensive molecular data for the entire cohort. Data were analyzed using descriptive statistics; results are presented as numbers (N) and percentages (%) for categorical and clinical variables, and as means for age and hemoglobin concentration to align with our descriptive approach. Clinical and biological baseline data were analyzed per patient (n=20), while bone marrow cytomorphological findings were analyzed per specimen (n=29). Given the exploratory nature of this study and the small sample size, inferential statistical analyses were not performed. All analyses were performed using Microsoft® Office Excel® (Microsoft Corporation, Redmond, Washington, United States).

## Results

We received a total of 2,062 bone marrow aspirate examinations during our study period. Among all these examinations, MDS was confirmed in 20 patients (0.96%) out of 29 suspected bone marrow samples. A male predominance was noted with 11 patients (55%), with a mean age of 61 years. More than half of the patients were over 60 years of age. The majority of requests, 11 cases (55%), originated from the Department of Internal Medicine. In our study, secondary MDS cases were excluded, which implies that no patient had received chemotherapy, radiotherapy, or bone marrow transplantation. From a clinical perspective, the primary manifestations were mainly characterized by anemia syndrome observed in 10 patients (50%), hemorrhagic syndrome in three patients (15%), infectious syndrome in three patients (15%), an association of anemia and hemorrhagic syndrome in two patients (10%), an association of anemia and tumoral syndrome (splenomegaly) in one patient (5%), and finally, only one patient (5%) presented with isolated tumoral syndrome (splenomegaly). Biologically, the CBC revealed anemia in 15 cases (83.33%) with a mean hemoglobin concentration of 7.92 g/dL. In all these patients, anemia was non-regenerative, macrocytic in four patients (22.22%), normocytic in six patients (33.33%), and microcytic in three patients (16.66%). Furthermore, thrombocytopenia was detected in 10 (55.55%), while the platelet count was normal in three (16.66%). As for neutropenia, it was observed in only eight patients (44.44%). Blood smear (BS) examination was performed in the majority of our patients (18, 90%). The results are detailed in Table 1.

**Table 1:** Distribution of cases according to blood smear results (n=20 patients; n=18 peripheral blood smear analyses available).

Lineage	Cytopenias	Bone Marrow Blasts (%)	Number of Cases N (%)
Single Lineage	Thrombopenia	No blasts	3 (15%)
Single Lineage	Anemia	No blasts	2 (10%)
Single Lineage	Anemia	2% - 4%	1 (5%)
Single Lineage	Anemia	5% - 19%	1 (5%)
Bilineage	Anemia and Neutropenia	No blasts	3 (15%)
Bilineage	Anemia and Neutropenia	2% - 4%	1 (5%)
Bilineage	Anemia and Neutropenia	5% - 19%	0 (0%)
Bilineage	Anemia and Thrombopenia	No blasts	2 (10%)
Bilineage	Anemia and Thrombopenia	2% - 4%	0 (0%)
Bilineage	Anemia and Thrombopenia	5% - 19%	1 (5%)
Trilineage	Anemia, Neutropenia, and Thrombopenia	No blasts	4 (20%)
Total	-	-	18 (90%)

Regarding the bone marrow evaluation, a total of 29 bone marrow aspirate smears obtained from our 20 patients were analyzed. In the majority of these smears (25 cases, 86.21%), the bone marrow aspirate was hypercellular (rich). Megakaryocytes were graded at 3+ in 17 smears (58.62%). Qualitative abnormalities and dysplastic features were present across all evaluated smears (29 cases, 100%).

​Dysplasia of the megakaryocytic lineage (dysmegakaryopoiesis) was detected in 25 (86.21%) bone marrow smears, manifested mainly by nuclear hypolobulation and the presence of micromegakaryocytes. Dysgranulopoiesis affected 26 smears (89.66%), with hypogranulation or degranulation observed in almost all of these cases, alongside abnormal chromatin condensation. Dyserythropoiesis was identified in 15 smears (51.72%) and was primarily characterized by cellular gigantism with nucleocytoplasmic maturation asynchrony, bi-nuclearity, and vacuolated cytoplasm. Upon cytochemical examination, Perls' stain revealed the presence of ring sideroblasts in two smears (10%).

​Regarding bone marrow blast percentages across the 29 samples, 19 smears (65.52%) showed a blast count below 5%, four (13.79%) had a count between 5% and 9%, and the remaining six (20.69%) presented with an excess of blasts between 10% and 19%. According to the results of a cytogenetic study by the conventional method, there was only one case (5%) of 5q- syndrome and one case (5%) of chromosome 7q deletion among the 13 studies (65%) performed on all patients [4]. According to our results, the IPSS-R score was calculated in only seven cases, of which five had a score between 5 and 6, one had a score between 3.5 and 4.5, and finally, one had a score between 2 and 3.

According to the 2022 WHO classification, the 20 patients followed in our study were distributed across the different diagnostic categories as detailed below in Table 2 [5].

**Table 2:** Distribution of cases according to the WHO 2022 classification.

WHO Classification 2022	Number of Cases (N %)
Myelodysplastic neoplasm with few blasts and unilineage dysplasia	1 (5%)
Myelodysplastic neoplasm with few blasts and multilineage dysplasia	7 (35%)
Myelodysplastic neoplasm with few blasts and ring sideroblasts	2 (10%)
Myelodysplastic neoplasm with isolated 5q deletion	1 (5%)
Myelodysplastic neoplasm with increased blasts, type 1 (MDS-IB1)	6 (30%)
Myelodysplastic neoplasm with increased blasts, type 2 (MDS-IB2)	3 (15%)
Total	20 (100%)

## Discussion

MDS are rare in general but common among hematological neoplasms [1]. According to statistics from the Düsseldorf Bone Marrow Registry, an annual incidence of four per 100,000 in the total population and 20 per 100,000 in people over 70 years of age has been documented [1]. In our context, epidemiological data from a study by Hicham et al. [3] reported an average frequency of 7.6 cases per year, which aligns with our findings of an annual incidence of 1.40% among malignant hematopathies. Regarding the demographic data of our series, a slight male predominance is noted, with a male-to-female ratio of 1.2. This result is consistent with several reported series [2,6,7], which similarly demonstrate a male-to-female ratio ranging from 1.12 to 1.58. While some studies, such as those by Bekadja et al. [8], have reported a slight female predominance, our findings align with the broader epidemiological data indicating that males are often more frequently affected by MDS. Furthermore, the age group most affected in our study (those over 60 years) is consistent with established international patterns [2,3,7,8]. Clinically, manifestations are generally vague and non-specific, which explains the complexity and delay in diagnosis. Up to 30% of patients are asymptomatic with incidental discovery. The main symptoms of MDS result from peripheral cytopenias, with anemia syndrome being the major manifestation, characterized by cutaneous-mucosal pallor, asthenia, and tachycardia. Hemorrhagic syndrome or infectious syndrome may also be observed. In general, tumoral syndrome is uncommon, and its presence suggests rather an intermediate form between myeloproliferative neoplasia (MPN) and MDS. Furthermore, autoimmune manifestations may also be present. These include systemic diseases, seronegative polyarthritis, atrophic polychondritis, systemic and cutaneous vasculitis, inflammatory colitis, organ-specific autoimmune diseases, neutrophilic dermatoses, and lymphoid hematopathies. It is noted that 10-20% of MDS are accompanied by such autoimmune manifestations [8-10].

In our series, the primary manifestations are represented mainly by anemia syndrome in 10 (50%) [2,3,6,11]. This is consistent with the literature data. Furthermore, only two patients presented with an autoimmune disorder at the time of diagnosis (Biermer disease and rheumatoid arthritis), representing a prevalence of only 10%.

The CBC demonstrated the presence of one or more peripheral cytopenias, while cytological analysis of the BS revealed specific features such as blast cells and characteristic cellular dysplasia. It is crucial to emphasize that newly diagnosed cytopenias mandate a thorough investigation to exclude other underlying etiologies. Anemia was a consistent finding in our cohort, observed in 83.33% of patients, which aligns with established literature where anemia occurs in 80-85% of cases [3,12]. This anemia is typically non-regenerative, normochromic, and either normocytic or macrocytic.

According to literature data, thrombocytopenia exists either in isolation or combined with other cytopenias in 20% of cases [13]. This is not confirmed by our data (10; 55.55%). Furthermore, neutropenia was detected in eight cases (44.44%), although this indicator is confirmed in the literature at 20%.

When examining the BS, MDS may be suspected on the basis of morphological abnormalities. Dysmyelopoiesis is a cytological term encompassing abnormalities observed in all three cell lineages. It also allows verification of whether cytopenias and blasts are present in order to establish classification according to the 2022 WHO criteria [5].

The signs of dysmyelopoiesis frequently observed in the results of BS examination, according to several authors referenced in the literature [14-16], are the observation of red blood cells with macrocytic characteristics, red blood cells with basophilic punctations, and circulating erythroblasts. As well as degranulation of neutrophils (highly specific), nuclear hyposegmentation (Pseudo-Pelger-Huët type), or hypersegmentation of neutrophils, and then the presence of micromegakaryocytes [17]. According to our data, dysplasia signs of the neutrophil lineage, especially of the hypogranulation type, are the most frequent sign. Regarding the quantitative results of the BS, cytopenia in one to three lineages (thrombocytopenia, anemia and thrombocytopenia, or pancytopenia) with the absence of circulating blasts is the most frequently identified abnormality in our study. The bone marrow aspirate represents the fundamental examination for establishing the diagnosis of MDS through analysis of quantitative abnormalities of myeloid precursors. This examination provides crucial information regarding bone marrow cellularity and the presence of blasts, as well as signs of dysmyelopoiesis [2,3,15-18]. Furthermore, it allows identification of ring sideroblasts through Perls staining, which is essential for MDS classification [5,14,18]. It also plays a key role in evaluating prognosis and developing an appropriate therapeutic management strategy. Moreover, this tool proves useful for the differential diagnosis of MDS. In general, MDS is characterized by a hypercellular or normocellular bone marrow [3], although a minority of patients (approximately 10%) [3,19] may present with bone marrow hypoplasia. In our study, we observed that the majority of bone marrow samples were rich in cellularity. Dysmyelopoiesis designates a cytological syndrome that encompasses all qualitative abnormalities likely to affect the three cell lineages: erythroid (dyserythropoiesis), granulocytic (dysgranulopoiesis), or megakaryocytic (dysmegakaryopoiesis) [13]. According to the WHO, dysmyelopoiesis is significant when it affects at least 10% of elements of a lineage following evaluation of a minimum of 200 elements, with the exception of the megakaryocytic lineage (30 elements) [1,14-19].

Signs of dyserythropoiesis are defined by nuclear abnormalities such as nuclear irregularity, detached nuclear fragments such as Howell-Jolly bodies, intranuclear bridges, karyorrhexis, and bi- and multinuclearity, as well as cytoplasmic abnormalities such as lacunar or lamellar cytoplasm and cytoplasmic vacuoles and basophilic punctations. There are also signs, such as macro-erythroblasts and megaloblasts. Perls' staining must be systematic to validate the amount of iron contained in hemosiderin in the form of blue/green granules identifiable under the microscope during cytological analysis of bone marrow slides. Iron-containing erythroblasts called sideroblasts must be quantified on at least 100 erythroblasts. Ring sideroblasts are defined by the presence of five or more iron granules in a perinuclear position in more than one-third of the nuclear circumference [1,13,15,16,18]. Dysgranulopoiesis is detected by the presence of abnormalities, notably of the nuclear type, namely hypo- or hypersegmentation of the nucleus, abnormal chromatin condensation, and pseudo-Pelger-Huet abnormalities. For cytoplasmic abnormalities, they are less frequent and essentially represent hyper- or hypogranulation and persistence of basophilia or the presence of Auer rods [15,16]. Furthermore, the abnormalities present in dysmegakaryopoiesis are micromegakaryocytes, multinucleated megakaryocytes, and hypolobated megakaryocytes [16]. Although these abnormalities are less frequent on bone marrow smears, they hold major diagnostic importance [1].

Dysmegakaryopoiesis and dysgranulopoiesis were identified in 87.45% in our study, while dyserythropoiesis was detected in 44.82%, which is also detected in a study conducted by Hamri et al. [20] However, our data do not agree with those of a study conducted by Bibi et al. [2], where dyserythropoiesis was predominant.

By definition, the percentage of blasts must be between 0 and 19%. Beyond this threshold, a diagnosis of AML must be established. Most often, the morphological appearance of blasts is classic, with medium size and a high nuclear-to-cytoplasmic ratio. The boundaries of the nuclei are often oval, with fine chromatin and a nucleolus. The cytoplasm appears basophilic; it contains no archoplasm. The presence of Auer bodies classifies MDS with increased blasts type 2 [17]. The majority of bone marrow samples in our study show a blast percentage below 5%, which appears compatible with the data by Bibi et al. [2].

In certain circumstances, a bone marrow biopsy (BMB) may be necessary. BMB allows diagnosis of hypoplastic MDS through assessment of cellular richness. This variety occurs at a frequency estimated at approximately 10% of MDS. Furthermore, BMB is the only means of accessing a diagnosis of bone marrow fibrosis [15,16], which makes it the diagnosis of MDS with fibrosis. Bone marrow fibrosis will be found in approximately 10% of MDS, depending on the authors [2]. No patient in our study was subjected to this examination. It is crucial to perform cytogenetic analysis of bone marrow cells if MDS is suspected to confirm the diagnosis and assess the prognosis of these diseases using the IPSS score [1,2,15-19]. Cytogenetically, MDS are highly heterogeneous. Primary MDS and secondary MDS are distinguished by the degree of karyotype complexity observed at the time of disease diagnosis: 30-60% of de novo MDS present an abnormal karyotype, while 90% of chemotherapy-induced MDS present abnormal karyotypes [2,13]. In our study, we excluded secondary MDS. The abnormalities identified in the various studies are diverse: numerous partial chromosomal deletion(s) and monosomies, by far the most frequent, while gains of chromosomes and balanced chromosomal translocations are rare [17,20,21]. The isolated and partial deletion of the long arm of chromosome 5 is the main abnormality observed, making it a distinct entity among MDS, the 5q- syndrome [17]. This syndrome essentially affects women and is generally of good prognosis, with a risk of transformation to AML of less than 10% [13,22-24]. It was detected in only one case in our study. The different classifications have evolved from the French-American-British (FAB) classification of 1976 to the most recent proposals of the fifth edition of the WHO classification, as well as the International Consensus Classification (ICC) of 2022 [23].

Following the 2022 WHO classification [5], the entity of MDS with few blasts and multilineage dysplasia (MDS-LB-MLD) was the most frequently detected in our study (seven, 35%), whereas most other studies are based on the 2008 WHO classification, in which the most frequently found type is refractory cytopenia with multilineage dysplasia (RCMD) [1-5,14,18,23,24]. This entity is characterized by the presence of one or more cytopenias associated with dysplasia of two or three lineages in the bone marrow. The proportion of blasts is less than 1% in peripheral blood and less than 5% in the bone marrow, without Auer bodies [24].

Patients with MDS have a heterogeneous prognosis. Therefore, it is essential to perform risk stratification in patients with myelodysplasia. Indeed, the IPSS is the most frequently used score to stratify the risk in patients suffering from myelodysplasia, but the tendency toward underestimation of risk related to the "Low" or "Intermediate -1" category necessitated a revision of the score, giving rise to the IPSS-R, increasingly used by clinicians and considered the best prognostic tool [1,4,24]. The IPSS-R provides a more granular prognostic assessment by incorporating five key clinical and laboratory variables: cytogenetic abnormalities, bone marrow blast percentage, hemoglobin level, platelet count, and absolute neutrophil count. By integrating these refined parameters, the IPSS-R distinguishes five distinct risk categories (ranging from 'Very Low' to 'Very High'), offering a more precise outcome prediction than its predecessor [2,4,13,25-28]. In our cohort, this score was calculated for seven patients who underwent a complete diagnostic workup, including successful cytogenetic analysis. Within this subgroup, five patients fell into the high-risk category (score 5-6), one into the intermediate-risk group (score 3.5-4.5), and one into the low-risk category (score 2-3). This distribution, characterized by a predominance of high-risk cases, suggests a potential referral bias toward patients with more severe symptoms necessitating extensive diagnostic evaluation. Given the aforementioned prognostic criteria, our prognostic stratification must be interpreted with caution. Since our study faced limitations regarding the availability of complete cytogenetic data for the entire cohort, these missing variables prevent a fully robust application of the IPSS-R score, thereby highlighting the necessity for a more systematic diagnostic workup in our future clinical practice to optimize risk assessment.

This study has several limitations that must be acknowledged. First, the sample size is relatively small (n=20), reflecting the single-center nature of our study. Second, due to the retrospective design, clinical outcome data were limited; while we documented one case of progression to AML, comprehensive long-term follow-up was constrained by patient attrition. Consequently, we could not provide a robust analysis of overall survival or treatment response for the entire study population. Furthermore, since conventional cytogenetic analysis (karyotyping) was performed in only 13 out of 20 patients due to local accessibility issues, a complete and rigorous prognostic stratification using the IPSS-R could not be established for the entire cohort. Moreover, the absence of comprehensive molecular testing, such as next-generation sequencing (NGS), represents a further limitation. As molecular mutations are increasingly pivotal for definitive diagnosis and precise prognostic stratification under the 2022 WHO classification, our reliance on morphological and cytogenetic data alone may lead to an underestimation of the molecular complexity of our MDS cases. While these limitations are inherent to our study design and regional context, it is crucial to address their implications. The small sample size and single-center nature of our research impose limitations on the generalizability of our findings. Furthermore, incomplete cytogenetic and molecular data, while reflecting real-world clinical accessibility challenges in our setting, may lead to an underrepresentation of specific high-risk MDS subtypes. Consequently, our findings should be interpreted as a foundational descriptive baseline for the Oriental region of Morocco rather than an exhaustive epidemiological assessment. Future multicenter studies, coupled with enhanced access to molecular diagnostic technologies, will be essential to validate these preliminary observations and strengthen the prognostic framework for MDS management in our clinical setting. Despite these limitations, our findings provide crucial baseline biological and cytomorphological insights into the management of MDS in the Oriental region of Morocco.

## Conclusions

MDS encompasses a panel of clonal malignant hematopathies involving the hematopoietic stem cell. Recently, particular attention has been paid to this pathology within the framework of diagnostic, prognostic, and treatment criteria to prevent its complications, with transformation into AML being the major example. In 2022, new MDS classifications emphasized the importance of genetic abnormalities in these pathologies; therefore, integrating these updated diagnostic criteria is essential.

However, implementing these advanced diagnostic standards in routine practice remains a challenge in resource-limited settings. Consequently, our study was constrained by several factors, including a limited number of patients (n=20), the rarity of accessible cytogenetic tests, and a lack of comprehensive molecular data.

Despite these limitations, this study provides a vital descriptive baseline profile of MDS in the Oriental region of Morocco, highlighting the practical application of the 2022 WHO classification in a real-world clinical setting. Our findings confirm that dysmyelopoiesis and cytopenias are the primary diagnostic features in our cohort. Given the retrospective, single-center nature of our work, these observations should be interpreted as preliminary and cannot be generalized to the broader population. Nevertheless, this work underscores the critical need for expanded access to standardized cytogenetic and molecular diagnostics to improve prognostic stratification and patient management. Our results serve as an initial step toward larger, multi-center prospective studies that will be essential to validate these findings and strengthen the diagnostic framework for MDS management in our region.
